# An Automatic Detection Method for Cutting Path of Chips in Wafer

**DOI:** 10.3390/mi14010059

**Published:** 2022-12-26

**Authors:** Yuezong Wang, Haoran Jia, Pengxuan Jia, Kexin Chen

**Affiliations:** Faculty of Materials and Manufacturing, Beijing University of Technology, Beijing 100124, China

**Keywords:** microscopic vision, visual inspection, chip, wafer cutting, interlayer

## Abstract

**Highlights:**

- Cutting path Planning System for wafer images without Mark points in different imaging states.
- Use the interlayer in the chip region as an auxiliary location to determine the cutting path.
- The determined cutting path is located in the middle of the streets, away from the chip region.

**Abstract:**

Microscopic imaging is easily affected by the strength of illumination, and the chip surface qualities of different wafers are different. Therefore, wafer images have defects such as uneven brightness distribution, obvious differences in chip region characteristics, etc., which affect the positioning accuracy of the wafer cutting path. For this reason, this thesis proposes an automatic chip-cutting path-planning method in the wafer image of the Glass Passivation Parts (GPPs) process without a mark. First, the wafer image is calibrated for brightness. Then, the template matching algorithm is used to determine the chip region and the center of gravity position of the chip region. We find the position of the geometric feature (interlayer) in the chip region, and the interlayer is used as an auxiliary location to determine the final cutting path. The experiment shows that the image quality can be improved, and chip region features can be highlighted when preprocessing the image with brightness calibration. The results show that the average deviation of the gravity coordinates of the chip region in the *x* direction is 2.82 pixels. We proceeded by finding the interlayer in the chip region, marking it with discrete points, and using the improved Random Sample Consensus (RANSAC) algorithm to remove the abnormal discrete points and fit the remaining discrete points. The average fitting error is 0.8 pixels, which is better than the least squares method (LSM). The cutting path location algorithm proposed in this paper can adapt to environmental brightness changes and different qualities of chips, accurately and quickly determine the cutting path, and improve the chip cutting yield.

## 1. Introduction

In the field of wafer cutting, the technology to determine the cutting path primarily includes mechanical structure positioning and microscopic image processing. The mechanical structure determines the wafer street by adjusting the spacing, which is simple and time-saving. However, the mechanical structure has low positioning accuracy and easily damages the chip due to positioning deviation [1]. Micro-image processing technology can find the wafer street and cutting path with high magnification, and the accuracy can reach the micrometer scale, but it requires high image quality and takes time. According to whether there is a Mark point or not, wafers are divided into two categories. When there is a Mark point on the wafer, the wafer’s attitude and street location information can be obtained by determining the position and rotation angle of the Mark point [2]. When there is no Mark, the wafer’s attitude and street location are determined by determining the position and attitude of the chip region in the image, and then the cutting path is determined. Correctly locating the position and attitude of the Mark point or chip region is the prerequisite for determining the cutting path. Its positioning accuracy determines the accuracy of the cutting path. The higher the accuracy, the less likely the chip will be damaged, and the higher the yield of the chip production.

The number of Mark points on the wafer is mostly one or two. We use the *x*, *y* translation stage to find the Mark points and determine the rotation angle and street location of the wafer according to the position and attitude of the Mark points [3,4]. When there is only one Mark point on the wafer, the rotation angle of the wafer is the same as that of the Mark point. When there are two Mark points, the angle between the connecting line between the two Mark points and the horizontal direction is used as the rotation angle of the wafer. We repeatedly measure the rotation angle of the wafer and straighten it until the rotation angle is less than a certain threshold, and the street location is determined by the location of the Mark point (the Mark point is located in the center of the block). When there is no Mark point in the wafer, the wafer rotation angle and block position are determined by the chip area, or directly determine the block position in the image. Common methods for detecting the wafer rotation angle and cutting path include the template matching algorithm [5], the contour edge detection algorithm [6,7,8,9,10,11], and the establishment of neural networks [12,13]. Qi et al. (2005) first smoothed the image, enhanced the image with histogram equalization, and finally determined the location of the chip region in the image with the Sum of Squared Differences (SSD) template matching algorithm. The edge detection algorithm mainly uses the Canny operator to extract the outline edge of the chip and uses robust statistics methods to eliminate outliers, reduces the impact of uneven edges on the chip location accuracy, and finally, uses a rectangle fitting algorithm to find the chip region 6 or obtain the maximum connected domain of the edge and calculate the four vertices of the detected rectangular box to determine the rotation angle of the wafer [8]. Wang and Zhang (2021) used a convolutional neural network to roughly align the wafer, with an error of ±5°. Regression analysis was used when the fine alignment was performed. Before fine alignment, brightness calibration was required for the image, and then the slight difference in feature points between the two images was compared to derive the rotation angle of the wafer. Yu et al. (2021) proposed an improved U-Net convolution neural network method to directly determine the cutting path. To speed up the process, the image is down-sampled to one-quarter of the original image, and the location of the chip location is determined using the advantageous genetic Adaptive Genetic Algorithm (AGA) for the downsampled image [14]. In contrast, the quadratic exponential function is used to smooth the histogram of the image and obtain the binary image, distinguish the street and chip region, and calculate the cutting path according to the binary result [15]. Current algorithms focus on finding the edges of chip regions and determining the chip region and Mark point locations using template matching algorithms, which only exploit the geometric features and grayscale variations of the image and have high requirements for image quality. The neural network algorithm has high accuracy in street recognition through training, but it requires a large number of images to train different specifications of wafers, which is time consuming.

In summary, the existing methods used to determine the cutting path based on the wafer image have the following problems: (1) The wafer images processed are all taken under a high magnification optical system and there is a great deal of pollution in the street and chip region. Although template matching and contour edge detection can determine the location of the chip region, it is very difficult to accurately determine its chip region with high accuracy. (2) When the neural network is used to find the street directly, it requires a lot of training and takes a long time. Moreover, the interference factors of the street region in each image are different, and the recognition accuracy is disturbed. (3) Only the contour features of the chip region in the image are used, and no other useful information about the chip region is used.

In order to solve the above problems, this paper proposes a cutting path planning algorithm for the chip region in the wafer image. First, the tilted wafer is aligned, and then the brightness calibration of the image is performed on a global scale. The chip region and the center of gravity position are determined by calculating the frequency domain correlation between the template and the target image, and the interlayer position is found in the determined chip region. The interlayer is used as an auxiliary to positioning to determine the cutting path. The accuracy of real-time performance and adaptability between different state images using the chip interlayer as the auxiliary location to determine the cutting path is verified by experiments. The innovation of our method is mainly reflected in three aspects: (1) The proposed image brightness calibration algorithm can improve image quality. Compared with the traditional image enhancement and grayscale transformation algorithms, when the illumination changes significantly, it can better improve the overall grayscale distribution of the image. (2) A template matching algorithm based on frequency domain correlation is used to find the chip region, which enhances the dependence on the frequency domain characteristics of the chip region and reduces the chip region pollution and other factors’ interference. (3) On the premise of determining the chip region, the interlayer is used as an auxiliary to positioning, and the cutting path is not affected by the geometric shape change of the chip region. The determined cutting path is distanced from the chip region to improve the yield of wafer cutting.

The remainder of this article is organized as follows: In Section 2, the materials and methods are designed; in Section 3, the experiments are carried out; in Section 4, the discussion is carried out. In Section 5, the conclusions are derived.

## 2. Materials and Methods

### 2.1. Wafer Cutting Principle

**Figure 1:**
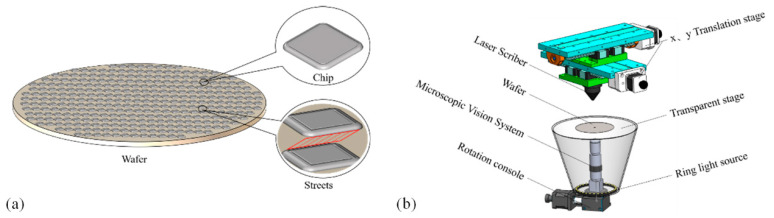
Wafer cutting principle. (**a**) Wafer model; (**b**) the composition of the wafer dicing machine.

Wafer cutting refers to using a cutter or laser to separate the chips arranged on the wafer and finally form a separate chip. With the iterative upgrade of production technology, to improve the utilization rate and production efficiency of wafers, reduce the spacing between chips, improve the arrangement density of chips, or reduce the size of chips, the chip size can reach hundreds of microns or even smaller, and larger-sized chips are on the millimeter level. As chips become smaller, streets become narrower, and chip spacing becomes closer, the possibility of chip damage during cutting becomes greater, so it is more important to accurately locate streets and provide the appropriate cutting path. The wafer-cutting principle is shown in Figure 1. Figure 1a shows the wafer model, the rectangular blocks arranged on the surface are chips, and the wafer street is defined as the red shaded area in the amplification area.

The wafer dicing machine is shown in Figure 1b. The dicing machine is composed of a rotation console, a microscopic vision system, a transparent stage, a ring light source, a laser scriber, an *x*, *y* translation stage, and a computer. The microscopic vision system is composed of a charge-coupled device (CCD) camera, an automatic zoom lens, and a large magnification metallographic objective lens. For chips of different sizes, the chips within the field of vision can be adjusted to an appropriate size by combining objectives of different magnifications and changing the magnification of the automatic zoom lens. The magnification of the microscopic vision system can be adjusted between 10 and 200 times.

According to the cutting principle of the dicing machine, the wafer is placed upside down on the stage, the microscopic vision system is adjusted to the appropriate magnification according to the size of the chip in the wafer, the captured wafer image is transmitted to the computer, and the computer calculates the wafer position and attitude. The DD motor adjusts the wafer position and attitude according to the calculation results. Calculation of the cutting path starts after the wafer has been aligned. During the processing, the image coordinate system needs to be converted to the spatial coordinate system. The *x*, *y* translation stage drives the laser to the designated position of the wafer for cutting.

### 2.2. Definition of Cutting Path

**Figure 2:**
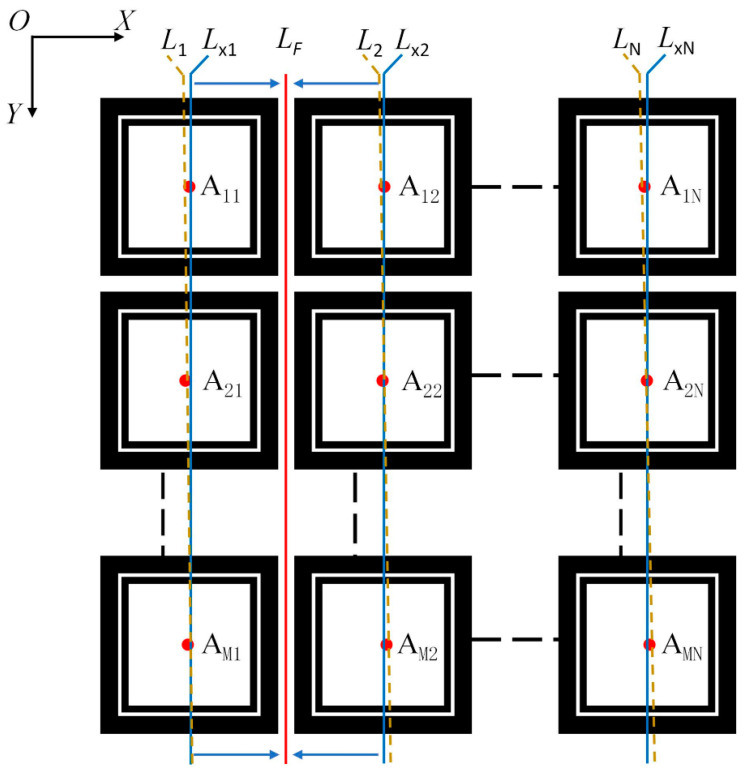
Definition of cutting path on the ideal wafer image.

Visual inspection is an important method to determine the wafer-cutting path. It requires that the cutter does not damage the chip when cutting along the cutting path. Therefore, in the process of visual solution, the positioning accuracy of the cutting path determines the yield of the final chip production. When cutting, it is considered that the chip pattern, geometric shape, and brightness distribution in the ideal wafer image are highly similar; otherwise, it is a real wafer image. To process an ideal wafer, it is only necessary to determine the center of gravity position of the chip region to determine the cutting path, which meets the accuracy requirements. The definition of the cutting path is shown in Figure 2. In Figure 2, the image coordinate system is *o*-*xy*, where *o* is the origin, *x* is horizontally positive to the right, and *y* is vertically positive in the downward direction. Assuming that the number of chips in the image is *M* (rows) × *N* (columns), the center of gravity position of each chip region *A_ij_*(*x_ij_*, *y_ij_*) was determined by the template matching algorithm of calculating the frequency domain correlation, in which *i* represents the row index and *j* represents the column index, the center of gravity position of each column of chips is fitted using the Least Square Method (LSM), and the fitting line *L_j_* of the column *j* is obtained. The equation of the straight line is *y_j_* = *a_j_x* + *b_j_*. We correct the fitting of the straight line. The slope of the corrected straight line is the same. The corrected straight line *L_xj_* of column *j* is obtained, and the equation is *y_xj_* = *a_x_* + *b_xj_*, where *a_x_* is the average slope of all fitted straight lines. Then, we determined the cutting path *L_F_* from the modified lines *L_x_*_1_ and *L_x_*_2_ on both sides of the first column of the street. The cutting path equation is *y_F_* = *a_Fx_* + *b_F_*, *a_F_* = *a_x_*, and *b_F_* is the mean of the intercept of the two straight lines.

**Figure 3:**
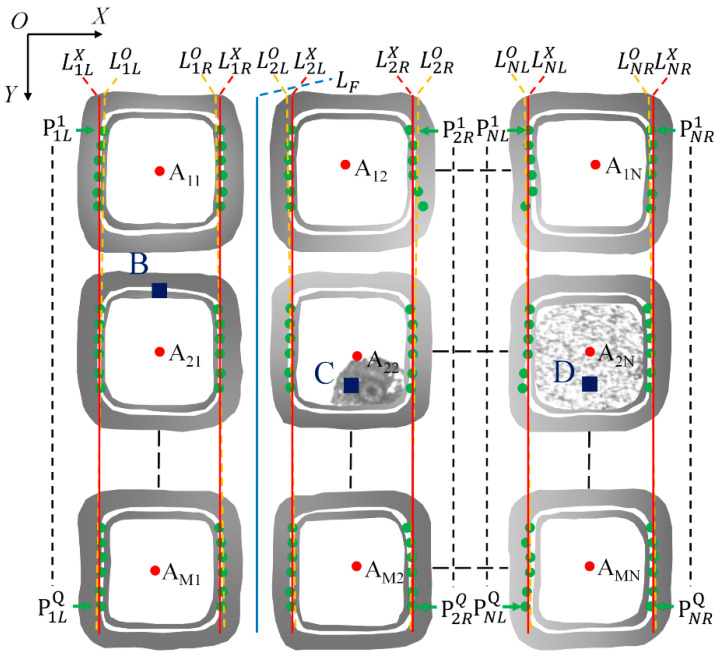
Definition of cutting path on the real wafer image.

When determining the cutting path of the real wafer image, the overall brightness distribution, geometric shape, and surface quality of the chip region are significantly different due to the influence of external light and wafer surface pollution, as shown in Figure 3. The brightness distribution in the *A_MN_* chip region in the image is uneven as the left side is bright and the right side is dark. The areas marked C and D represent surface contamination in the chip region.

Based on the above analysis, due to the influence of light and surface pollution, the positioning deviation of the chip region is more than 10 pixels, or even higher. Therefore, it is necessary to preprocess the image, adjust the gray distribution, and highlight the chip region. After the chip region is determined, the interlayer is used as an auxiliary to positioning to determine the cutting path. The trace is shown in the area marked *B* in Figure 3. In the real case, the template matching algorithm is used to determine the center of gravity *A_ij_* of the chip region, which is the same as the ideal positioning method. The peak detection algorithm is used to find the interlayer in the determined chip region, and discrete points are used to mark it. The discrete points are divided into $P_{jL}^{q}$ and $P_{jR}^{q}$ according to the left and right sides of the chip in column *j*, as shown by the green dot in Figure 3. We used the ANSAC algorithm [16] to fit the discrete points on each column of chips to obtain the straight line $L_{jL}^{O}$ and $L_{jR}^{O}$, corrected to $L_{jL}^{X}$ and $L_{jR}^{X}$ according to the correction line on both sides of the first street $L_{1R}^{X}$ and $L_{2L}^{X}$, to determine the cutting path *L_F_*.

### 2.3. Automatic Detection Algorithm of Chip Cutting Path

**Figure 4:**
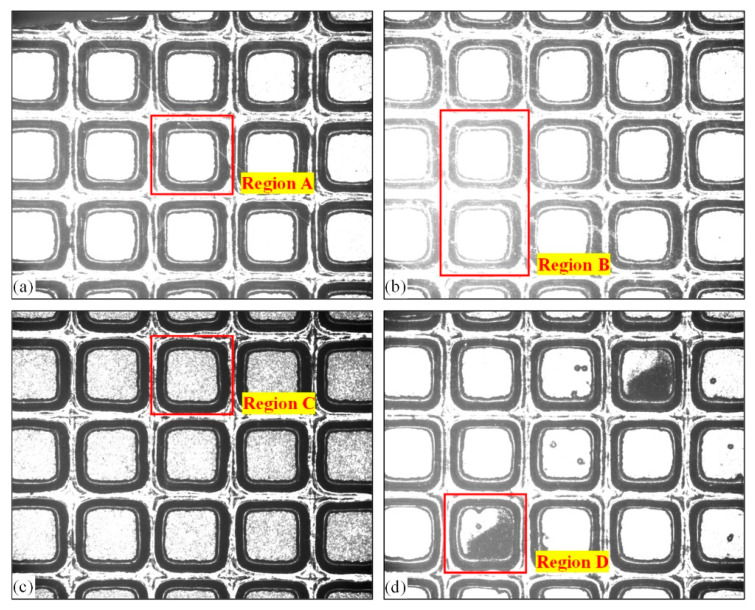
Classification of wafer images. (**a**) Wafer images classified as category 1; (**b**) wafer images classified as category 2; (**c**) wafer images classified as category 3; (**d**) wafer images classified as category 4.

The real wafer image has defects such as uneven brightness distribution in the chip region, inconsistent geometric shape, and pollution in the chip region and the street. The image in Figure 4 is defined and classified according to the defects in the chip region.

Figure 4a has the highest proportion of images in different categories. Compared with other categories of images, all chip regions in the image have uniform gray distribution and obvious interlayers, which are classified as qualified images. Region A in Figure 4a is a good chip region. Figure 4b is classified as an overexposed image. The image is generally bright, and there is a defect of uneven gray distribution in some chip regions. In Figure 4b, region B differs greatly from the area on the right. The chip contour is poorly differentiated from the street, and the interlayer is not obvious. Figure 4c is classified as too dark, as the image is generally dark and some chip regions appear as black particles, as shown in the region marked C in Figure 4c. Figure 4d is defined as an image with surface contamination. Individual chips are marked by ink dots because they fail to pass the electrical test or the photolithographic etching process is unqualified, resulting in a large area of black spots on the chip surface. When the above multiple chip regions appear in the wafer image, the processing method is the same. In Figure 4, regardless of what category the image is, there is pollution in the street. The shape of the chip region is roughly the same, but there is slight or obvious deformation in the interlayer, such as region D in Figure 4d.

**Figure 5:**
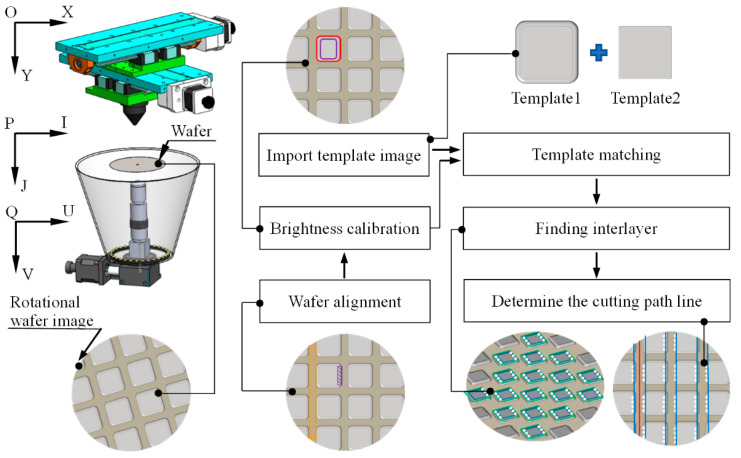
An algorithm for determining the cutting path of wafer image.

In order to adapt to the above four categories of wafer images and locate the cutting path accurately and quickly, the cutting path positioning algorithm is divided into four steps: Wafer alignment, image brightness calibration, chip region positioning, and cutting path determination. The algorithm flow is shown in Figure 5. The coordinate systems in Figure 5 are the space coordinate system *p*-*ij* where the wafer is located, the image coordinate system *q*-*uv,* and the motion coordinate system *o*-*xy*. To accurately determine the spatial position of the wafer through the micro vision system and make the laser cutter move to the starting position of the cutting path and move along the cutting path, it is necessary to calibrate the coordinate system and adjust the three coordinate systems to be parallel to each other and in the same direction. After the coordinate system is calibrated, we used the method proposed by a previous study [17] to align the rotating wafer. The brightness of the aligned wafer image is calibrated to improve the gray distribution of the image, highlight the chip region, and improve the image quality. Based on the brightness calibration, we selected the chip matching template and the template that limits the size of the area where the interlayer is located (the area-limiting template only serves to determine the area where the chip interlayer is located and does not perform any image processing). Then, the chip region and the center of gravity position (only one template is selected for wafers of the same specification) were determined by the template matching algorithm to calculate the frequency domain correlation, and then the area where the chip interlayer is located was determined by the region limit template to reduce the search range and improve the accuracy. We found the location of the interlayer in the determined interlayer area and used discrete point markers, as shown by the white discrete points in Figure 5. By fitting the discrete points and modifying the fitting line (averaging the slope of the fitting line and refitting the discrete points based on the slope determination) using the improved RANSAC algorithm, the cutting path was determined by a modified straight line on either side of the first street, with the cutting path being the midline of the two fitted lines, as shown by the blue line in Figure 5.

#### 2.3.1. Wafer Alignment

The front end of the slicing equipment is an automatic loading system. After the wafer is placed on the stage, it is located above the microscopic system. At this time, there are three coordinate systems in the system, as shown in Figure 5, which are the space coordinate system *p*-*ij* where the wafer is located, the coordinate system *o*-*xy* of the motion translation stage, and the image coordinate system *q*-*uv*. Before image processing, the three coordinate systems need to be aligned. Generally, the method of “inter-axis alignment” is used to keep the corresponding coordinate axes of the three coordinate systems parallel. After alignment, the plane attitude of the wafer is always consistent with the coordinate axes of the image coordinate system. When the laser cutter moves to the translation stage, the motion direction is always consistent with the coordinate axes of the image coordinate system.

**Figure 6:**
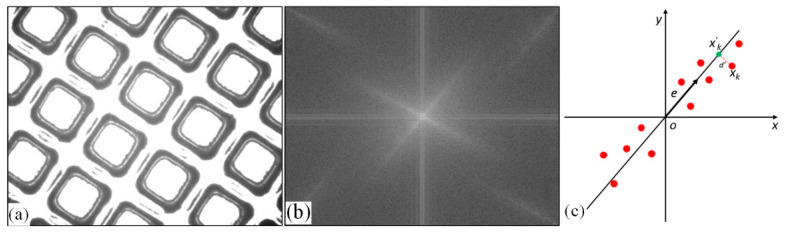
Wafer alignment. (**a**) Rotational wafer image; (**b**) amplitude spectrum; (**c**) discrete points fitting schematic.

For the wafer attitude problem, we used the method proposed by Xu et al. to achieve wafer alignment. When the image is regular in a certain direction, after two-dimensional Fourier transformation, the amplitude spectrum appears to have a larger peak in this direction, resulting in two mutually perpendicular lines in the amplitude spectrum. The chips in the wafer image are regularly arranged, and the amplitude spectrum has a large peak in this direction. As shown in Figure 6a, the wafer image is not aligned, and Figure 6b shows the amplitude spectrum after the two-dimensional Fourier transform. Using this property, a linear detection method based on the Hough transform was used [18]. First, the Hough transform is used to filter the points in the amplitude spectrum that are distanced from the peak line. After filtering, there are many scattered points around the line, and the square sum of the distance from the point to the fitting line is the smallest. The discrete points around the line are defined as {*x*_1_, *x*_2_, …, *x_N_*}. According to the properties of the Fourier transform, the detected peak line will pass through the center of the image. The center of the amplitude spectrum is defined as the origin of the image. We assume that the unit direction vector of the fitting line is *e*, ‖e=1‖, the distance from *x_k_* to the line is *d*′, the projection on the line is $x_{k}^{\prime }$, and the distance from $x_{k}^{\prime }$ to the origin *o* is *a_k_*, as shown in Figure 6c, in which the discrete points fit the schematic.

*J* is the standard function of square error, and the unknown parameters are *a*_1_, *a*_2_, …, *a_N_* and *e*. Based on the above assumption, when the error is the smallest, the direction of *e* is the direction of the fitting line.

(1) $$J\left(a_{1},\ldots ,a_{n},e\right)=\overset{N}{\underset{k=1}{\sum }}{\left\|(x_{k}^{\prime }-x_{k})\right\|}^{2}\;$$

where $x_{k}^{\prime }=a_{k}e$, $a_{k}=e^{t}x_{k}$. We can extend (*a*) to

(2) $$J\left(e\right)=-\overset{N}{\underset{k=1}{\sum }}{\left[e^{t}x_{k}\right]}^{2}+\overset{N}{\underset{k=1}{\sum }}\|x_{k}\|{}^{2}$$

*S* is defined as

(3) $$S=\sum_{k=1}^{N}e^{t}x_{k}x_{k}^{t}e$$

According to Equations (2) and (3)

(4) $$J\left(e\right)=-e^{t}Se+\overset{N}{\underset{k=1}{\sum }}\|x_{k}\|{}^{2}$$

When *J* is the minimum value, −*e^t^Se* is the maximum value, and the maximum value is at the point where the first derivative is equal to 0. The Lagrange multiplier is introduced to solve −*e^t^Se*, and *S* is a scatter matrix that can be solved by the eigenvalue. Finally, we solved the equation and found the direction vector of the fitting line. The included angle between the fitting line and the *x*-axis is the angle that the wafer needs to be squared.

#### 2.3.2. Wafer Image Brightness Calibration

Due to the influence of the strength of illumination and chip category, different categories of chip surfaces reflect different intensities of the incident light, resulting in differences in the brightness of different chip regions. The images are divided into four categories, as shown in Figure 4. For the stereotypical algorithm, processing images with different brightness will output different detection results, while wafer cutting requires strict precision of the cutting path. In the algorithm, the template matching method is used to search for the center of gravity of the chip region in the image. After the template used for matching is determined, the grayscale distribution and frequency domain characteristics of the template image remain unchanged throughout the detection process. When matching images with excessive differences, such as the A area in Figure 4b, it is easy to output the wrong matching results. In addition, when searching for the interlayer, it is necessary to highlight the interlayer area, so as to ensure the stability of the interlayer search. Therefore, it is very important to calibrate the brightness of wafer images in different categories to improve the gray distribution and highlight the feature areas.

In order to calibrate the brightness difference between different batches of images, it is necessary to adjust the overall gray distribution of the image, rather than in a specific, small range of gray intervals. Therefore, it uses the exponential transformation in the nonlinear image enhancement algorithm to transform the image gray level, enhance the contrast between the chip region and the street, and highlight the interlayer [19]. The grayscale transformation rules of the exponential method are as follows:

(5) $$I\left(m,n\right)=f{\left(m,n\right)}^{a}$$

*f*(*m*, *n*) represents the gray value of the pixel at row *m* and column *n* in the target image, and the image size is *M* × *N*. *I*(*m*, *n*) is the gray value of the corresponding part after exponential calculation, and *a* is the exponential parameter. The selection of *a* is related to the overall gray distribution of the image. The value of *a* is:

(6) $$a=\{\begin{array}{c} e^{c1\times Level}-d_{1}\;Level\geq T_{d}\\ e^{c2\times \sqrt{Level}}-d_{2}\;Level<T_{d}\; \end{array}$$

where *Level* is the global threshold value of the image gray value, which is between 0 and 1. It is obtained by employing the Maximum Between-Class Variance Method [20]. *Td* is the evaluation threshold value for judging over darkness and overexposure, and *c*_1_, *c*_2_, *d*_1_, and *d*_2_ are constants. When the *Level* is greater than *Td*, the image brightness will be reduced. When the *Level* is less than *Td*, the image brightness will be increased. The value of *Level* is:

(7) $$Level=\arg\underset{k}{\max}\left(\sigma_{k}\right)$$

*σ_k_* is the between-class variance calculated when the gray value is *k*:

(8) $$\sigma_{k}=\omega_{k0}\omega_{k1}{\left(\mu_{k0}-\mu \right)}^{2}$$

*K* is greater than or equal to 0 and less than 256. When the pixel value is *k*, the area whose gray value is less than *k* is defined as the foreground, and the area whose gray value is greater than *k* is defined as the background. At this time, the number of pixels in the foreground area is *N_k_*_0_ and the number of pixels in the background area is *N_k_*_1_, where *ω_k_*_0_ = *N_k_*_0_/*(M* × *N)*, *ω_k_*_1_ = *N_k_*_1_/(*M* × *N*), and *µ_k_*_0_ are the average gray values of the foreground area when the gray value is *k*, and *μ* is the average gray value of the image.

#### 2.3.3. Automatic Detection of Chip Region

At present, the most commonly used method to determine the chip position is the template matching algorithm. Template matching algorithms are based on the gray value [21], correlation [22], and geometric shape [23]. Among them, the gray-scale correlation matching method is more suitable for scenes with stable lighting. Although the correlation matching method can adapt to lighting, large errors will occur when the target area is deformed. Considering the difference in the shapes of different chips, the shape of the chip region in some images changes greatly and is irregular, and sometimes the street will be connected to the chip region and affect the matching accuracy of the geometric shape.

The wafer image contains multiple chip regions. In the same batch of wafer products, the chip appearance is roughly the same, but there are also differences. In order to accurately match all chip regions and center-of-gravity positions, we used a template matching algorithm based on frequency domain analysis. The selection of matching templates is described in detail in Section 3.1, and all features of the chip region should be included as much as possible.

The template matching algorithm based on the computational frequency domain correlation includes the following steps:

**Step 1**: Parametric settings for the target image and template image.

**Figure 7:**
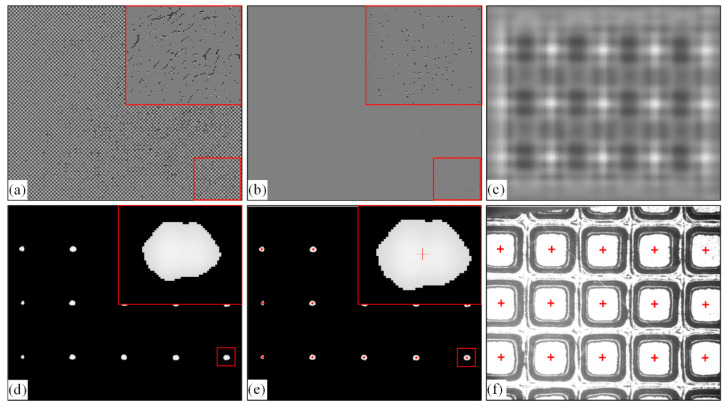
Detection results of the chip region for each step. (**a**) The result of the parameterized template; (**b**) the result of the parameterized wafer image; (**c**) correlation result; (**d**) the result of thresholding; (**e**) the result of extremum search; (**f**) the center of gravity position of the chip region.

We assume that the size of target image *I* is *M* × *N*. The size of the selected template image *T* is *m* × *n*. The target image *I* and the template image *T* rotated by 180 ° are changed to the frequency domain space by Fourier and are expanded to the size of (*M* + *m* − 1, *N* + *n* − 1). The parameterization equation is as follows:

(9) FI=K(ℱ(I),(M+m−1,N+n−1))

(10) FT=K(ℱ(rot(T,180)),(M+m−1,N+n−1))

where *K*(*I*, (*m*, *n*)) represents the extended function, expanding *m* rows and *n* columns, respectively, in the row and column directions of *I*, using trailing 0 to fill. *rot*(*I*, *θ*) indicates that *I* is rotated counterclockwise *θ*. *F* represents the Fourier transform. The processing results are shown in Figure 7a,b, and the lower right corner area is locally enlarged.

**Step 2**: Calculate the correlation between the parameterized wafer and the template image, and obtain the correlation matrix *R*.

Each value in the correlation matrix *R* represents the similarity between the sub-image at the corresponding position in the target image and the template image. The processing result is shown in Figure 7c. The correlation matrix *R* is expressed as:

(11) $$R=0.5+\frac{Icoor-meanIT}{2\times stdT\times P}$$

*Icoor* is the real part of the inverse Fourier transform results of the multiplication of the corresponding elements of *FI* and *FT*.

(12) $$Icoor={ℱ}^{-1}{\left(FI\left(x,y\right)\times FT\left(x,y\right)\right)}_{R}$$

where ${ℱ}^{-1}$ represents the inverse Fourier transform and *meanIT* is defined as the average compensation:

(13) $$meanIT=\frac{L\left(I,\left(m,n\right)\right)\times \sum_{i=1}^{m}\sum_{j=1}^{n}T\left(i,j\right)}{m\times n}$$

*L*(*I*, (*m*, *n*)) adds *I* to *m* rows and *n* columns and calculates the row-wise cumulative sum based on the column-wise cumulative sum of *I.* The *stdT* and *P* in Equation (11) are defined as:

(14) $$stdT=\sqrt{\left(m\times n-1\right)\times \frac{\sum_{i=1}^{m}\sum_{j=1}^{n}\left(T\left(i,j\right)-\bar{T}\right)}{m\times n}}$$

(15) $$P=\max\left(\sqrt{\frac{\max\left(L\left(I\times I,\left(m,n\right)\right)-\frac{L{\left(I,\left(m,n\right)\right)}^{2}}{m\times n},0\right)}{m\times n}},\frac{stdT}{100,000}\right)$$

where $\bar{T}$ is the mean of *T*, and max (•) is defined as the maximum value of the corresponding element selected by comparing a matrix with a matrix or a matrix with a constant. Calculating the frequency domain correlation between the template image and the target image avoids traversing every sub-image in the image, and only processes the frequency spectrum of the template image and the target image, avoiding a great deal of calculation.

**Step 3**: Non-Maximum Suppression (NMS) is used to process the correlation matrix *R* to determine the position and region of the chip’s center of gravity.

The correlation matrix *R* was processed by NMS [24] to obtain the location *A_ij_* with the highest similarity in each region. In the correlation matrix *R*, the larger the gray value is, the greater the possibility that this position is the center of gravity of the chip region, and the area with a low gray value is distanced from the chip region. Therefore, we set the area in the correlation matrix where the gray value is lower than the threshold *T_d_*_1_ to 0, determined the region where each chip is located, as shown in Figure 7d, and then took the position of the maximum value in each area as the center of gravity of the chip region, as shown in Figure 7e. We determined that the maximum value position in the correlation matrix of the thresholding process is (*x_i_*, *y_i_*), used the red cross mark, determined the search radius $r_{0}=\sqrt{m^{2}+n^{2}}$ around the maximum value position, set all gray values within the radius r0 around the maximum value to 0, and then repeated the process until all regions had been searched to obtain the center of gravity positions of all chips in the target image, as shown in Figure 7f. The size of the chip region in the image can be determined by the size of the template. The range and center of gravity of the chip region were used to find the interlayer in the next section.

#### 2.3.4. Determination of Cutting Path

Because the template matching result inevitably has a deviation from the center of gravity of the chip region, when the deviation exceeds the safety threshold, the laser cutter will damage the chip when cutting the wafer along the cutting path determined by the matching result. However, the interlayer is still in the matching area, so the street can be located according to the interlayer, and then the cutting path can be determined. In order to solve the impact of template matching deviation, we found the location of the interlayer in the determined chip region. The interlayer is the white area marked with B in Figure 3. There is an obvious gray level change between the interlayer and the adjacent area. When the gray level changes from left to right or from right to left, the gray level value of the pixel closer to the interlayer is larger and the change is more intense. According to this feature, the interlayer positions on the left and right sides of the chip area are determined.

According to the obvious gray-level change on both sides of the interlayer, when the gray-level value suddenly changes dramatically in the search process, this position is considered the edge of the interlayer. In order to improve the stability of searching for the interlayer, when searching for the interlayer, the sum of the gray values of ten vertically adjacent pixels was taken as the evaluation value of peak detection, and then the interlayer position was determined according to the area where the interlayer is located, as determined in the previous section. In order to eliminate the influence of pixel value fluctuation, when the difference between the peak and valley is greater than 1, it is considered an effective peak. The first effective peak is located at the edge of the interlayer and marked with discrete points, as shown by the green discrete points in Figure 3. The discrete points on the left side of the chip are marked as $P_{jL}^{q}$ and the right discrete point is marked as $P_{jR}^{q}$. Q represents the number of discrete points and *j* represents the number of columns of the chip.

The cutting path is determined by the marked discrete points. The improved RANSAC algorithm is used to fit the discrete points. During the fitting process, the fitting line will be constantly modified to remove the abnormal discrete points and reduce the influence of incorrect judgment of the interlayer. The RANSAC algorithm is shown in Formula (16):

(16) $$L=ransac\left(\left\{p_{1},\cdots ,p_{Q}\right\},\rho ,d,\alpha \right)$$

where *L* is the determined fitting straight line, {*p*_1_, …, *p_Q_*} is *q* discrete points to be fitted, *ρ* is the number of discrete points randomly selected for fitting, and it is considered that when the distance between the discrete point and the fitting straight line exceeds *d*, it is an abnormal point. *α* is the proportion of assumed normal discrete points in the data, and assuming that the fitting straight line needs to be iterated *k* times, the probability of at least one abnormal point at the selected point during fitting is 1 − *α^ρ^* and the probability of randomly selected p points as normal points is:

(17) $$z=1-{\left(1-\alpha^{\rho }\right)}^{k}$$

According to Equation (17), the number of iterations is:

(18) $$k=\frac{\log\left(1-z\right)}{\log\left(1-\alpha^{\rho }\right)}\;$$

In iteration *k*, we select the fitting line with the least abnormal points as the final fitting result, and the fitting on the left side of the column *j* of chip regions is:

(19) $$L_{jL}^{O}:y_{jL}^{O}=a_{jL}^{O}x+b_{jL}^{O}$$

The right fitting line is defined as $L_{jR}^{O}:y_{jR}^{O}=a_{jR}^{O}x+b_{jR}^{O}$, as shown by the orange dotted line in Figure 3. Because the fitting lines on both sides of the street should be parallel when determining the cutting path, it is necessary to modify the fitting lines so that the slopes are equal. Based on determining the slopes, we used Equation (16) to refit the discrete points. The modified lines are $L_{jL}^{X}:y_{jL}^{X}=\alpha x+b_{jL}^{X}$ and $L_{jR}^{X}:y_{jR}^{X}=\alpha x+b_{jR}^{X}$, as shown by the red line in Figure 3, where *α* is the average slope of the unmodified fitting line. We located the cutting path in the first street, and the cutting path equation is $L_{F}:y_{F}=\alpha x+b$, as shown by the blue line in Figure 3, where:

(20) $$b=\frac{b_{1R}^{X}+b_{2L}^{X}}{2}$$

**Figure 8:**
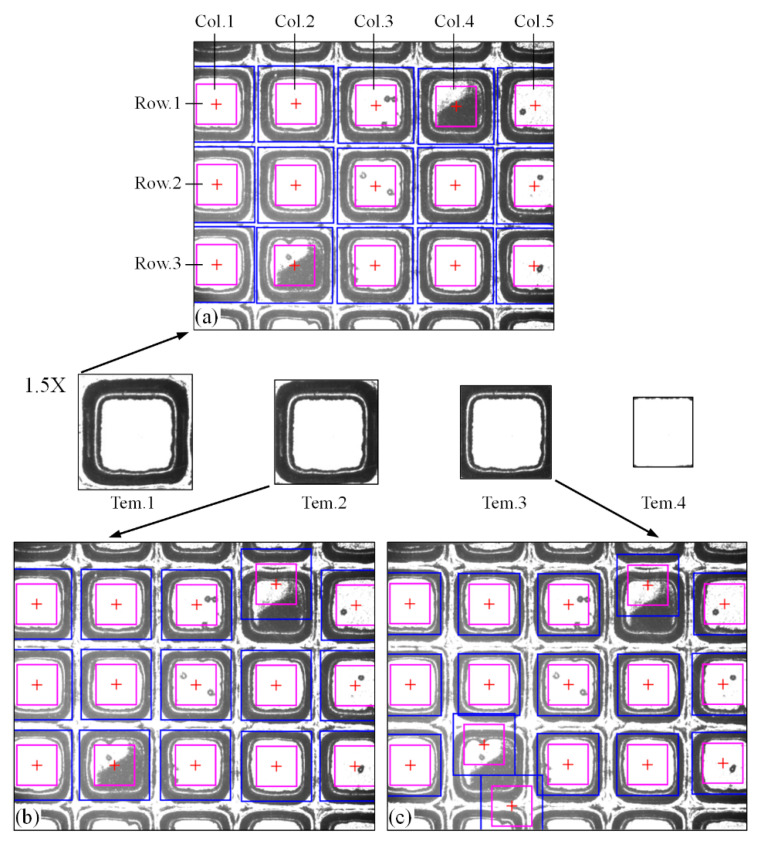
Comparison of template matching results for different templates. (**a**) Matching result of Tem.3; (**b**) matching result of Tem.2; (**c**) matching result of Tem.1.

Because the search for the cutting path is based on the interlayer, the determined cutting path will be distanced from the chip region, and the chip will not be damaged during cutting. Figure 8 shows the processing results of the real wafer image. It can be seen that the modified fitting line (yellow line) is located at the edge of the interlayer, and the determined cutting path (red line) is located in the center of the street, away from the chip area.

## 3. Results

In this section, the effect of brightness calibration is analyzed through experiments to determine the position accuracy of the chip area and the accuracy of the cutting path. The system in Figure 1b is used in the experiment. The magnification of the optical system is known to be 50×, and the actual chip size is 35 × 35 μm, the camera resolution is 1024 × 1280, and the size in the chip image coordinate system is 250 × 250 pixels.

The experiment randomly adopted 30 images of Category 1, Category 2, and Category 3, and 10 images of Category 4. Table 1 shows 10 images selected from the previous three categories and 5 images selected from Category 4.

### 3.1. Comparison of Template Selection

When the chip region is seriously polluted, templates of different sizes will produce different matching results. In order to compare the matching effects, three templates of different sizes are selected in a chip region, and a limit template Tem.4 is used to determine the range of the interlayer. Cat.4_1 in Table 1 is selected for the test image. The matching result is shown in Figure 8.

Figure 8 shows that two contaminated chip regions are successfully matched using the Tem.1 template, the incomplete chip regions on both sides of the image are also successfully located, and the interlayer is within the determined search range (the area between the blue frame and the pink frame is the search range of the interlayer). Tem.2 matches the lower contaminated area, and the upper contaminated area matches incorrectly. Tem.3 matched incorrectly in both contaminated chip regions, where the pixel sizes of Tem.1, Tem.2, Tem.3, and Tem.4 are 270 × 270, 250 × 250, 225 × 225, and 169 × 143, respectively. The pixel size of the chip area to be matched is 250 × 250. Therefore, when some street areas are covered around the selected template, the matching accuracy can be improved.

**Figure 9:**
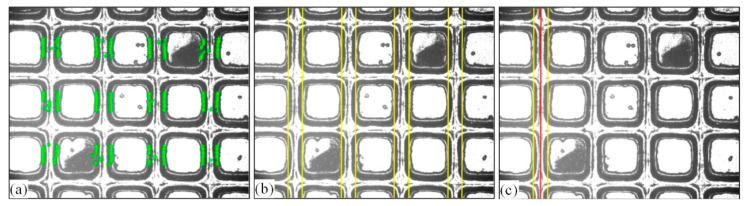
The result of each step of the cutting path. (**a**) The interlayer in the chip region; (**b**) straight lines; (**c**) cutting path.

Template Tem.1 is used to calculate the cutting path of image Cat4.1, the determined interlayer position (marked with discrete points), the fitted straight line, and the finally determined cutting path, as shown in Figure 9. Due to the pollution in the street, there are discrete points that are wrongly located in the polluted area, but they are removed during fitting. The determined fitting lines are close to the interlayer, and the cutting path is located in the center of the first street, far away from the chip region, which can ensure that the chip will not be damaged during cutting.

Table 2 summarizes the *x*-coordinate of the center of gravity of the chip region matched by using Tem.1 and calculates the deviation of the *x*-coordinate of each column of the chip. The position of the chip region is located in rows and columns. The matching deviation of each column is less than or equal to 5 pixels. It is normal in the conclusion in Section 3.3 and meets the use requirements.

Table 3 shows the MAE (Mean Absolute Error) evaluation results of the fitted straight line on the left and right sides of each column of chip regions. The evaluation results are all within 2 pixels, 87.5% of the results are within 1.5 pixels, and the average error is 1.24 pixels, achieving a good fitting effect. The evaluation results show that the improved RANSAC algorithm effectively removes discrete points.

### 3.2. Evaluation of Brightness Calibration

**Figure 10:**
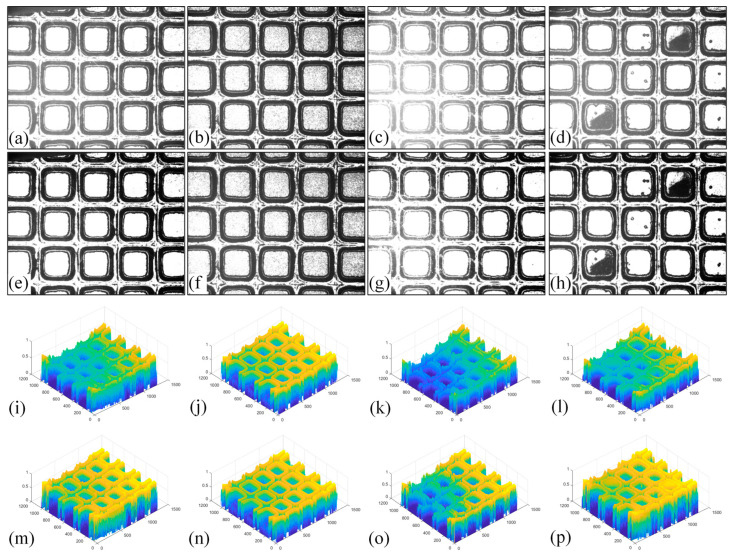
Comparison of brightness calibration for four categories of wafer images. (**a**) The wafer image of Cat.1_7; (**b**) the wafer image of Cat.3_7; (**c**) the wafer image of Cat.2_7; (**d**) the wafer image of Cat.4_1; (**e**) the result of brightness calibration on (**a**); (**f**) the result of brightness calibration on (**b**); (**g**) the result of brightness calibration on (**c**); (**h**) the result of brightness calibration on (**d**); (**i**) the grayscale distribution map of (**a**); (**j**) the grayscale distribution map of (**b**); (**k**) the grayscale distribution map of (**c**); (**l**) the grayscale distribution map of (**d**); (**m**) the grayscale distribution map of (**e**); (**n**) the grayscale distribution map of (**f**); (**o**) the grayscale distribution map of (**g**); (**p**) the grayscale distribution map of (**h**).

Experiments used 100 images for brightness calibration, and the results were analyzed qualitatively and quantitatively. By directly observing the contrast between the chip and the street in the wafer image before and after the brightness calibration, we observed whether the wafers in different categories are similar after the brightness calibration. Except for special circumstances, the gray distribution of the chip region should be similar. Figure 10 shows the contrast of image brightness before and after calibration in different categories.

By directly observing the wafer image before and after brightness calibration and the gray distribution map, it can be found that the chip region in the image after brightness calibration is more prominent, more distinct from the street, and the gray distribution is roughly the same, so the matched chip position will be more stable. The brightness of the wafer image in category 3 is not significantly improved after the calibration, and the black patches in the chip region of the image in category 4 are enhanced.

In order to verify whether the image quality improved after calibration, Brisque (BQ) and Niqe (NQ) [25,26], two non-reference image quality evaluation functions, are used for quantitative analysis of images before and after calibration. The smaller the evaluation values of the two methods, the better the image quality. The comparison of NQ and BQ quality before and after the brightness calibration of wafer images in different categories is shown in Table 4.

As shown in Table 4a,b,g,h, since the brightness of the image of Cat.1 and Cat.4 is uniform, it is easy to distinguish between the background area and the feature area, so the image quality is significantly improved. As shown in Table 4c,d, when the NQ evaluation function evaluates the image of Cat.2, the image quality is not significantly improved, while when the BQ evaluation function evaluates the image of Cat.2, the quality of the fourth to ninth images is not significantly improved. This is because, in an overexposed environment, the background and feature areas are relatively close, and pixel values are clustered in a small range, which cannot effectively improve the image quality. From Table 4e,f, we can see that in the images of Cat.3, the image quality has only slightly improved. Since 82% of the image quality increased after brightness calibration, 18% of the image quality did not improve. In a too-dark environment, the background area and feature area of different images are different, and the quality is also uneven, which makes it difficult to improve image quality. Since most of the wafer images of Cat.1 are collected during wafer cutting, and only a small part of the wafer images of other categories are collected, brightness calibration can effectively enhance the characteristic area of the chip and adapt to different brightness environments.

### 3.3. Analysis of Positioning Error in the Chip Region

The brightness difference of wafer images, defects in chip regions, shape changes, and other factors have a great impact on the matching results of the chip. We selected 100 wafer images proposed in the paper to evaluate the error of the matching algorithm. Because the chip position in each wafer image is not fixed, the final matching result is used to count the error. In Figure 3, we define the matching error *σ_j_* of the chip in column *j*:

(21) $$\sigma_{j}=\max\left\{A_{1j}\left(x\right),A_{2j}\left(x\right),\cdots ,A_{Mj}\left(x\right)\right\}-\min\left\{A_{1j}\left(x\right),A_{2j}\left(x\right),\ldots ,A_{Mj}\left(x\right)\right\}$$

*σ_j_* is the difference between the maximum value and the minimum value of the *x*-coordinate of the wafer in column *j*. The error should be greater than or equal to the error in the *x* direction between the calculated matching result and the accurate position when the exact position of the chip is known.

Table 5 shows the average error of images after brightness calibration. The average matching error of the wafer images of cat.1, cat.2, and cat.4 is within 3 pixels. The average error of the image of cat.3 is 3.36 pixels because of two outliers. An error of fewer than 4 pixels accounts for 94% of the total image, and an error of more than 5 pixels accounts for only 2% of the total image. Finally, the average error of all images in the x direction is 2.82 pixels.

**Figure 11:**
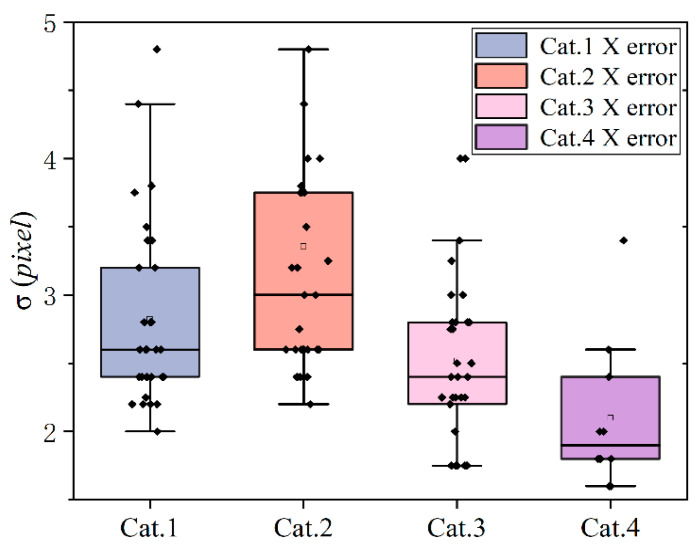
Error evaluation for template matching.

As shown in Figure 11, the accuracy of the enhanced image is mostly stable within 4 pixels, and only 2% of the images have a large positioning error deviation, which is greater than 5 pixels. All of them appear in the too-dark image, but the deviation is less than 8 pixels. The center of gravity and area of the chip obtained by template matching are prepared for finding the interlayer. This experiment proves that the matching accuracy obtained can determine the area where the interlayer is located, even when the matching deviation is 8 pixels, and it also meets the requirements for determining the area where the interlayer is located.

### 3.4. Accuracy and Stability Evaluation of Cutting Path

**Figure 12:**
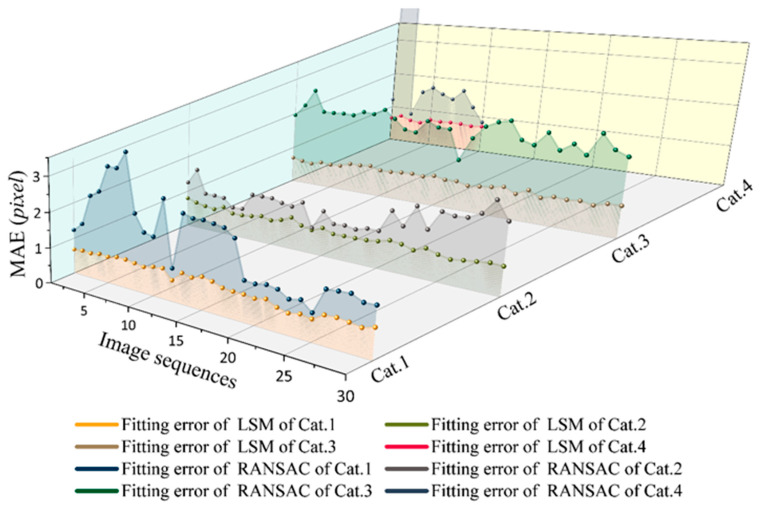
Comparison of fitting error between LSM and RANSAC algorithms for four categories of wafer images, where LSM is Least Squares Method.

This section verifies that the fitting accuracy of the improved RANSAC is better than that of the LSM through experiments. The higher the fitting accuracy is, the more accurate the position of the interlayer is, and the closer the final positioning cutting path is to the ideal position. The experiment used 100 images. We use the Mean Absolute Error (*MAE*) [27] to evaluate the fitting accuracy of cut paths in images.

(22) $$MAE=\frac{\sum_{i=1}^{m}\left|YReal-YPred\right|}{m}$$

where *m* is the number of discrete points, *YReal* is the real coordinate value, and *YPred* is the predicted coordinate value. The smaller the calculated MAE value, the higher the fitting accuracy. The average value of the MAE of the fitting line in the image is taken as the fitting accuracy of this image. As shown in Figure 12, different categories of images use RANSAC and the LSM to fit the precision comparison of discrete points.

It can be seen from Figure 12 that the fitting accuracy of the improved RANSAC is higher than that of LSM, and the fitting accuracy of RANSAC is stable. The MAE value evaluated fluctuates within 0.5–0.9 pixels, with little fluctuation. LSM fluctuates greatly due to the influence of abnormal points. When processing the Cat4 image, an exception occurs. The deviation distance is up to 20.18 pixels, which will locate the wrong cutting path. When the distance between the discrete point and the fitting straight line is less than 1 pixel, it indicates that the influence of the abnormal discrete point is removed, the reserved discrete point can be accurately positioned to the interlayer, and the fitting straight line is tangential to the interlayer. The cutting path located by the fitting straight line will be distanced from the chip and located in the center of the street, ensuring that the chip will not be damaged during the cutting process.

## 4. Discussion

To verify the accuracy and stability of the template matching algorithm used in the paper, we compare it with SAD and NCC matching algorithms, evaluate the number of chips matched, matching deviation, and running time, and verify the adaptability, accuracy, and real-time of the template matching algorithm. The images in Section 3.1 are used as experimental samples. When the deviation exceeds 10 pixels, the area where the interlayer is located cannot be guaranteed due to variations in the shape of the chip, resulting in cutting tracks with deviations in position and angle.

The Sum of Absolute Differences (*SAD*) traverses the entire target image and calculates the absolute difference between the sub-image and the template image. The smaller the absolute difference, the more similar it is.

(23) $$SAD\left(i,j\right)=\sum_{s=1}^{M}\overset{N}{\underset{t=1}{\sum }}\left|S\left(i+s-1,j+t-1\right)-T\left(s,t\right)\right|$$

where, *S* is the target image and *T* is the template image.

The Normalized Cross Correlation (*NCC*) is similar to the SAD in that it uses the gray level of the target image and template image to calculate the matching degree by the normalized correlation measure.

(24) $$NCC\left(i,j\right)=\frac{\sum_{s=1}^{M}\sum_{t=1}^{N}\left|S^{i,j}\left(s,t\right)-E\left(S^{i,j}\right)\right|\cdot \left|T\left(s,t\right)-E\left(T\right)\right|}{\sqrt{\sum_{s=1}^{M}\sum_{t=1}^{N}{\left[S^{i,j}\left(s,t\right)-E\left(S^{i,j}\right)\right]}^{2}\cdot \sum_{s=1}^{M}\sum_{t=1}^{N}{\left[T\left(s,t\right)-E\left(T\right)\right]}^{2}}}$$

where *E*(*S^i^*^,*j*^) and *E*(*T*) represent the average gray value of the search area and template at (*i*, *j*), respectively.

To ensure the accuracy of the street location, when column *j* in the wafer image locates only one chip, it is considered that this column does not match the chip because the deviation of the cut path determined by only one chip is large. The adaptability of the algorithm is determined by processing the number of chips matched in different wafer images and the matching accuracy rate. The more chips are located, the better the adaptability of the method. The definition of accuracy is the same as that in Section 3.1. The difference between the maximum and minimum value in the *x*-direction coordinate position of each chip column is obtained, and then the mean value of the difference in each column of an image is calculated as the positioning error of the image. The smaller the average difference, the more accurate the positioning is. Real-time is defined as the average processing time for each wafer image in different categories.

Table 6 shows the comparison of the number of SAD, NCC, and proposed method matching chips with the correct rate, deviation in the X direction, and processing time.

If too few chips are matched, there will not be enough chips on either side of the street to locate the cut path. Table 6 shows that the matching accuracy of the proposed method is the highest, the matching results are all correct, and the number of chip regions matched in the wafer images of Cat.2, Cat.3, and Cat.4 are the largest. Although the SAD matches the largest number of chips in the image of Cat.2, the accuracy is less than 90%, and as the number of matches between this method and SAD is less than 1, the NCC method has the worst effect. In terms of accuracy, when using the proposed method to process images of Cat1 and Ca2, the deviation in the X direction is the smallest. Among the image processing results of Cat3 and Cat4, the deviation using the SAD is the smallest, but the number of chips matched by the SAD is also less than that of the proposed method. In terms of real-time, the running time of the proposed method is stable within 0.7 s, while the speed of processing the image of Cat.4 is slightly slower and stable at 1.1 s. In the experiments in Section 3.1, the fitting accuracy of Cat.4 is the best. The processing time of SAD and NCC algorithms is 44.5 s and 195 s, respectively. This is because the selected template is large and the traditional method needs to traverse the target image to obtain the evaluation value of each position, so the processing speed is slow and does not meet the real-time requirements. The proposed method is several times or even hundreds of times faster than the traditional method. It should be noted that the processing time of this method is the time to determine the cutting path, while the two traditional methods only match the time.

## 5. Conclusions

The cutting path planning of wafer images without Mark points proposes a cutting path localization algorithm using the interlayer as an auxiliary location. In the calculation process, the grayscale distribution rule and self-characteristics of the image are fully considered. Due to the factors of the working environment and wafer quality, the grayscale distribution of the image is not uniform, and the interlayer is not prominent. Therefore, the image is pretreated by brightness calibration. By analyzing the frequency domain characteristics of the chip region, the chip position is determined by using a template matching algorithm based on frequency domain correlation calculation, and the wafer cutting path is determined by searching for the interlayer. Through the positioning experiment of the cutting path, the following conclusions are obtained:

(1) Through brightness calibration of wafer images in different categories, the gray distribution of different wafer images is roughly the same, highlighting the frequency domain and interlayer of the chip region, improving the quality of images and the accuracy and stability of matching results.
(2) The experimental results show that the template matching algorithm based on frequency domain correlation is better than the traditional template matching algorithm in terms of matching accuracy, stability, and real-time performance.
(3) Removing the abnormal discrete points marking the position of the interlayer can improve the positioning accuracy of the cutting path. Before and after removal, the fitting accuracy is increased by approximately 150%, and the slope of the fitting line is more stable.
(4) Using interlayers as an auxiliary location can reduce the impact of chip positioning results. The determination of the cutting path by interlayers on both sides of the street ensures that the determined cutting path is located near the center line of the street and away from the area where the chip is located.

For wafers of different specifications, one must select the corresponding template once. For different categories of wafer images, the corresponding cutting path planning algorithm can be designed, and a more detailed classification of wafer images can be carried out. In addition, the calculation time of the cutting path can be further reduced by reducing the search area of the interlayer.

## Figures and Tables

**Table 1:** Part of the wafer images.

Category 1	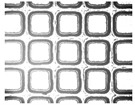	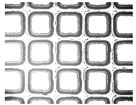	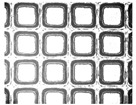	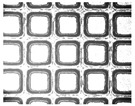	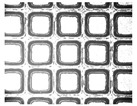
	Cat.1_1	Cat.1_2	Cat.1_3	Cat.1_4	Cat.1_5
	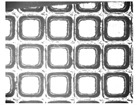	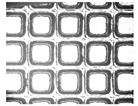	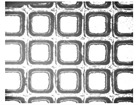	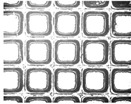	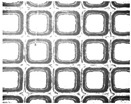
	Cat.1_6	Cat.1_7	Cat.1_8	Cat.1_9	Cat.1_10
Category 2	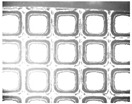	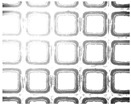	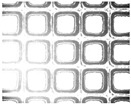	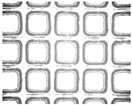	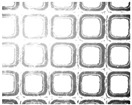
	Cat.2_1	Cat.2_2	Cat.2_3	Cat.2_4	Cat.2_5
	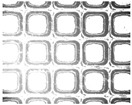	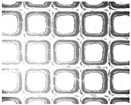	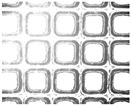	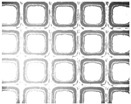	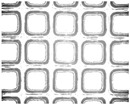
	Cat.2_6	Cat.2_7	Cat.2_8	Cat.2_9	Cat.2_10
Category 3	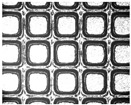	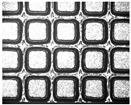	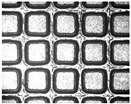	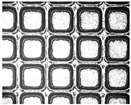	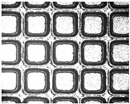
	Cat.3_1	Cat.3_2	Cat.3_3	Cat.3_4	Cat.3_5
	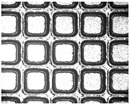	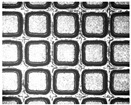	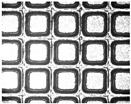	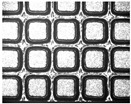	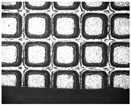
	Cat.3_6	Cat.3_7	Cat.3_8	Cat.3_9	Cat.3_10
Category 4	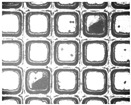	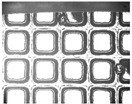	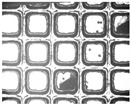	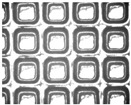	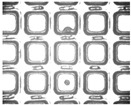
	Cat.4_1	Cat.4_2	Cat.4_3	Cat.4_4	Cat.4_5

**Table 2:** Matching chip *x*-coordinate and *x*-coordinate deviation per column (unit: Pixel).

	Index-Col	Col.1	Col.2	Col.	Col.4	Col.5
Index-Row						
Row.1		78	362	647	933	1216
Row.2		80	361	645	931	1213
Row.3		79	357	644	931	1211
X error		2	5	3	2	5

**Table 3:** Discrete point fitting MAE evaluation (unit: Pixel).

Chip	Chip2_L	Chip3_L	Chip4_L	Chip5_L	Chip1_R	Chip2_R	Chip3_R	Chip4_R
MAE value	1.692	0.9710	1.3562	1.1613	1.2513	0.8575	1.2527	1.4083

**Table 4:** The quality evaluation results of NQ and BQ before and after the brightness calibration for four categories of wafer images.

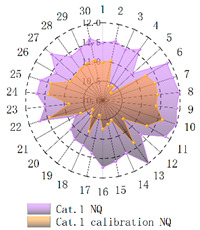	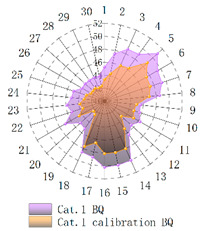	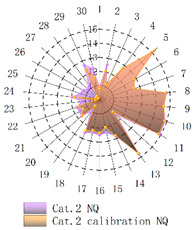
(**a**) NQ evaluation value before and after brightness calibration of Cat.1.	(**b**) BQ evaluation value before and after brightness calibration of Cat.1.	(**c**) NQ evaluation value before and after brightness calibration of Cat.2.
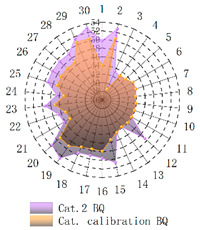	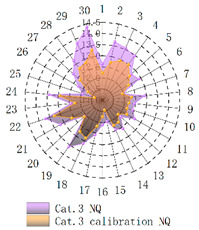	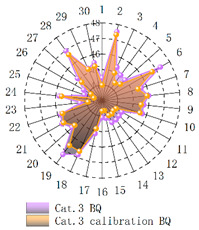
(**d**) BQ evaluation value before and after brightness calibration of Cat.2.	(**e**) NQ evaluation value before and after brightness calibration of Cat.3.	(**f**) BQ evaluation value before and after brightness calibration of Cat.3.
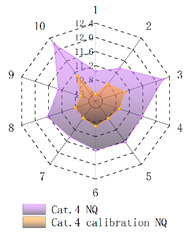	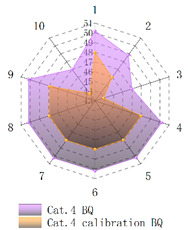	
(**g**) NQ evaluation value before and after brightness calibration of Cat.4.	(**h**) BQ evaluation value before and after brightness calibration of Cat.4.	

**Table 5:** Summary of image chip matching error after brightness calibration (unit: Pixel).

	Image	Cat.1	Cat.2	Cat.3	Cat.4
Error					
Mean error		2.82	3.36	2.52	2.10
≤2		1	0	7	7
2 < *σ* ≤ 3		20	26	19	2
3 < *σ* ≤ 4		7	10	4	1
4 < *σ* ≤ 5		2	2	0	0
*σ* > 5		0	2	0	0

**Table 6:** Comparison of the number and accuracy of matching chips, *x*-direction deviation, and processing time of SAD, NCC, and the proposed method.

Indicator	Chip Number/Relative Accuracy			*x*-Direction Deviation (Pixel)			Processing Time (s)		
Method	SAD	NCC	OUR	SAD	NCC	OUR	SAD	NCC	OUR
Cat.1	8.9/100%	11.0/86.7%	13.9/100%	3.24	7.92	2.82	44.2	194.0	0.65
Cat.2	5.7/100%	18.4/30%	13.6/100%	2.58	8.57	3.36	44.6	197.5	0.60
Cat.3	10.6/86.7%	6.33/20%	9.8/100%	6.91	7.67	2.52	44.9	197.5	0.64
Cat.4	7.0/100%	10.0/10%	13.1/100%	1.4	10	2.1	46.0	196.9	1.10
